# Unexpected Internal Jugular Vein Agenesis During Central Venous Cannulation: Implications for Ultrasound-Guided Access

**DOI:** 10.7759/cureus.108453

**Published:** 2026-05-07

**Authors:** Ashraf Abdelhalim, Joy Patel, Rajeev Dalal

**Affiliations:** 1 Anesthesiology and Perioperative Medicine, Penn State Health Milton S. Hershey Medical Center, Hershey, USA; 2 Anesthesiology and Perioperative Medicine, WellSpan Health, Lebanon, USA; 3 Anesthesiology and Perioperative Medicine, South Jersey Anesthesia and Pain Physicians, Inspira Health, Mullica Hill, USA

**Keywords:** central venous catheterization (cvc), internal jugular vein (ijv), jugular vein agenesis, ultrasound guidance, vascular anomaly

## Abstract

The right internal jugular vein (IJV) is the preferred site for central venous access due to its predictable anatomy and direct course to the superior vena cava. Congenital agenesis of the IJV is exceedingly rare and may have significant implications during central venous cannulation. We present a 67-year-old male patient undergoing hepatic resection who required central venous access after failure of peripheral intravenous lines. Pre-procedural ultrasound of the right neck demonstrated the absence of the right IJV despite appropriate positioning. Further examination confirmed right IJV agenesis and compensatory dilation of the left IJV. Given the necessity of central access and hemodynamic monitoring, a multidisciplinary discussion was undertaken regarding the potential risks of cannulating the sole major cerebral venous outflow pathway. The left IJV was successfully cannulated under ultrasound guidance without complication. Postoperative imaging reconfirmed the absence of the right IJV. This case underscores the importance of routine ultrasound evaluation prior to central venous cannulation. Recognition of rare vascular anomalies such as unilateral IJV agenesis is critical to prevent arterial injury and to guide safe anesthetic decision-making.

## Introduction

The internal jugular vein (IJV) is a bilateral structure arising in the posterior cranial fossa as a continuation of the sigmoid sinus. Its function is to permit the drainage of venous blood from the brain, regions of the face, and neck and descends inferiorly within the carotid sheath, along with the internal carotid artery and vagus nerve, before merging with the subclavian vein to form the brachiocephalic vein [1]. It is a common site for central venous cannulation given its superficial location and lack of variation in the majority of the population. Multiple indications for perioperative central line placement exist, such as large volume of fluid or blood product administration, measurement of hemodynamic values such as central venous pressures or pulmonary capillary wedge pressure, difficulty obtaining peripheral access, and many more. Ultrasound is commonly used by healthcare professionals as it improves the success rate and reduces the risk of complications.

The right IJV is commonly used for central venous cannulation by anesthesiologists due to its large diameter and has a less tortuous, straight path to the superior vena cava as opposed to the left IJV. The left IJV also tends to be slightly smaller than the right [1]. Cannulating the left IJV is associated with a higher risk of complications, including injury to the thoracic duct [2]. Vascular anomalies of major cervical vessels are rare but clinically significant. Developmental venous anomalies have been reported to be 0.05% to 0.25% [3], although rates as high as 20% have been reported for cerebral venous abnormalities in patients with cervical or facial venous malformations [4]. A review of the literature shows that only six cases of IJV agenesis have been published since 2010 [3,5-9]. Other vascular malformations of the IJV that have been reported include partial or complete duplication, stenosis, complete occlusion, distortions, and various intraluminal structures (i.e., membranes, webs, inverted valves) [10-15]. Here, we report our incidental finding of right IJV agenesis. Additionally, we corroborate the reported findings of contralateral distention of the left external jugular vein in the context of right IJV absence.

## Case presentation

A 67-year-old male patient with a past medical history of myocardial infarction status postpercutaneous coronary intervention on dual antiplatelet therapy (DAPT), coronary artery disease, hypertension, type II non-insulin dependent diabetes, class I obesity (BMI 34), treated hepatitis C with newly found hepatocellular carcinoma in segment seven, was scheduled for a diagnostic laparoscopy, exploratory laparotomy, cholecystectomy, and partial segment seven. The patient was a former IV drug user and had a history of difficult IV placement. He had IVs placed in his lower extremity behind the knee for previous procedures. He had an ultrasound-guided IV placed in the preoperative area, given his history. He was brought to the operating room, and standard ASA monitors were placed. After induction, it was noted that his peripheral access began to infiltrate. He was mask ventilated and ultimately successfully intubated, while another provider attempted to place peripheral access under ultrasound guidance. Unfortunately, these efforts were futile, and given the need for intravenous access and hemodynamic monitoring, including central venous pressure for the procedure, the decision was made to place a right-sided central venous catheter.

**Figure 1:**
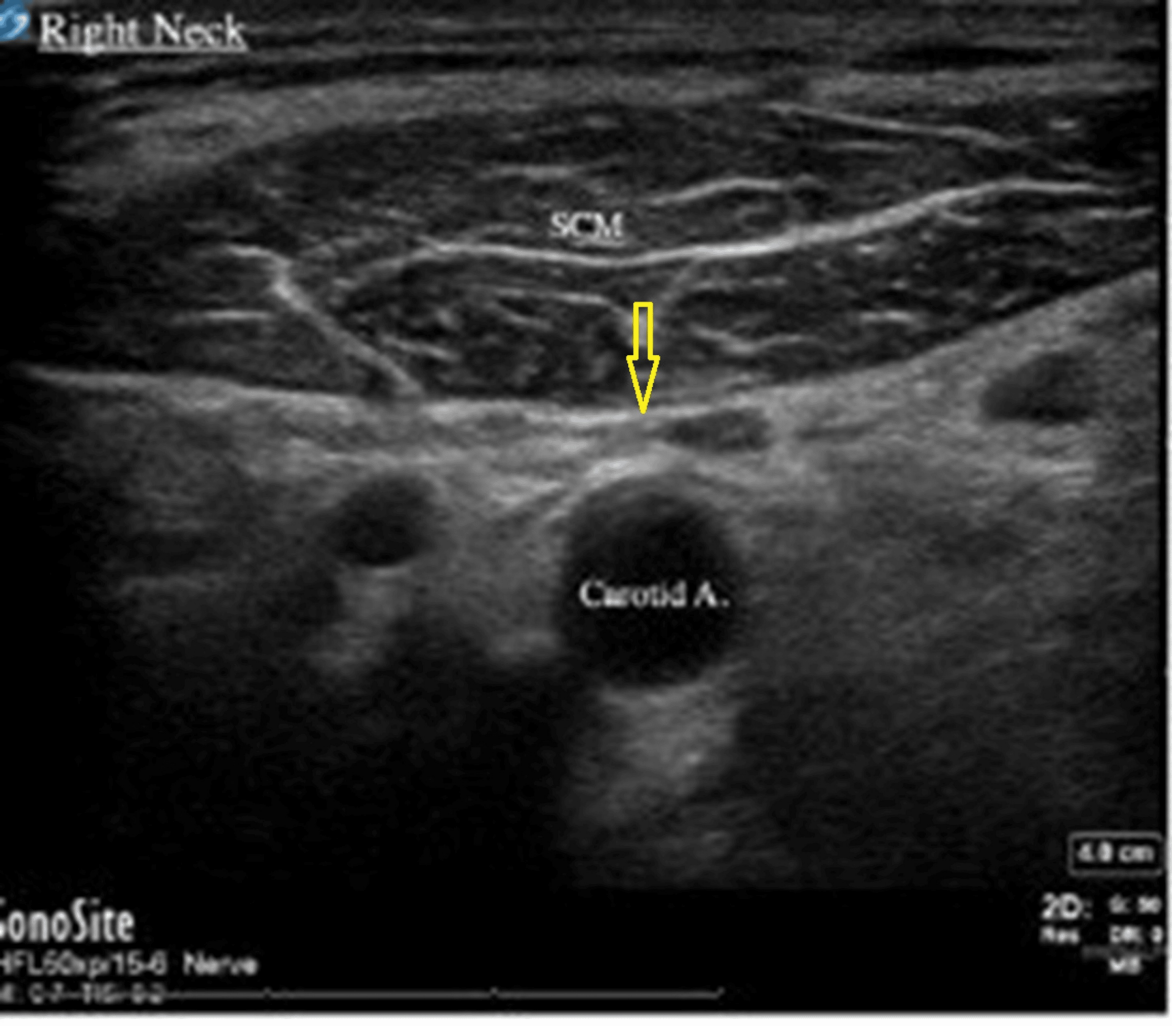
Ultrasound image of the right side of the neck showing yellow arrow at the expected site of internal jugular vein

The neck anatomy was assessed prior to venipuncture, and an unusually small caliber vein was identified while in the Trendelenburg position. Since this procedure was scheduled for the afternoon, it was hypothesized that the unusual size of the right IJV was due to hypovolemia from the patient being nil per os and proceeded with right-sided central venous cannulation. The neck was repositioned slightly and sterilized appropriately. Several attempts were made by both the anesthesia resident and anesthesia attending, but the IJV was unable to be visualized (Figure 1).

**Figure 2:**
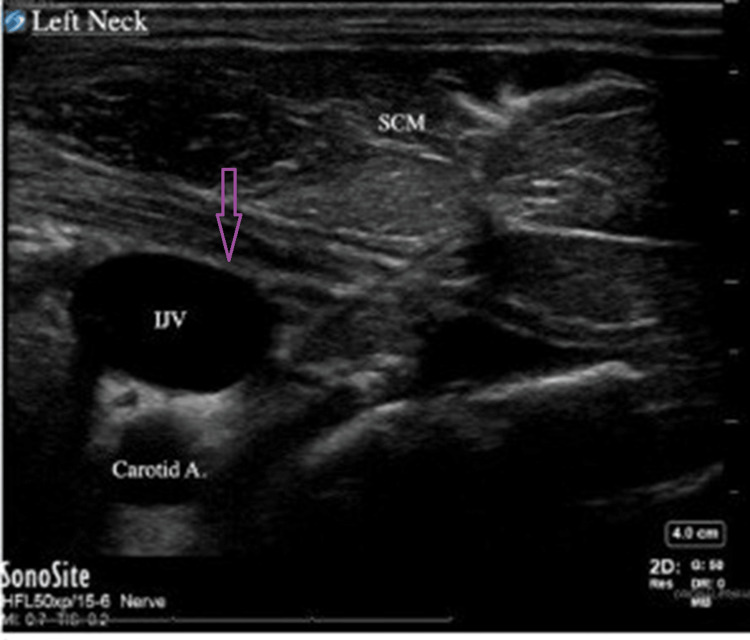
Ultrasound image of the left side of the neck showing purple arrow directed to IJV IJV: internal jugular vein

An experienced cardiac anesthesiologist was consulted to confirm such findings, and the anesthesia team collectively agreed that the right IJV was not present. The left IJV was then scanned with the ultrasound and was found to be overly distended (Figure 2). The anesthesiologist and surgeon had a risk vs benefit discussion regarding the need for access and potential complications that could arise from failed left IJV cannulation, including thrombosis, which would compromise venous return from the brain. The left IJV was prepped and successfully cannulated with no complications. The case was otherwise uncomplicated, and the patient was extubated.

These intraoperative findings were then discussed with the patient on postoperative day one. Images were not saved intraoperatively, so the patient gave verbal consent to use ultrasound and obtain these images. Written consent was also obtained in order to present these findings to the medical community. The images taken postoperatively occurred while the patient was in an upright position, so findings were slightly different from those in the Trendelenburg position intraoperatively; however, the right IJV absence was reconfirmed.

## Discussion

Given the ubiquity of right IJV cannulation for central venous access perioperatively and in the intensive care setting, providers should be aware of vascular anomalies they might encounter to minimize patient harm. Congenital absence of the right IJV is a rare anomaly, and it typically does not present significant clinical consequences [3]. A review of the literature shows that only six cases of IJV agenesis have been published since 2010 [5-9]. However, it highlights the necessity of performing a focused ultrasound examination in each patient prior to cannulation of a major venous structure. Early detection of vascular anomalies affords the clinician the opportunity to take appropriate precautions with regard to anesthetic management, cannulation site selection, and hemodynamic monitoring. In individuals with an absent right IJV, lack of awareness of such anomalies can readily lead to inadvertent cannulation of the carotid artery, especially when confounded by suboptimal patient positioning on the operating table or emergent placement underneath surgical drapes. Alternatively, cannulating the left IJV vein in the setting of an absent right IJV poses its own unique consequences [7]. Firstly, as described in the literature, the left IJV was readily identified as it was engorged, given its role as a sole venous channel for cerebral drainage. Injuring and compromising this sole structure during cannulation can have serious consequences, even if cannulation is performed with all precautions. Cannulation poses a chance of thrombosis of the left IJV and disruption of the venous drainage, potentially leading to increased intracranial tension secondary to venous congestion and cerebral edema, which can be life-threatening. Finally, this report contributes to the importance of routine precannulation ultrasound evaluation to identify rare but clinically significant vascular anomalies and to guide safe anesthetic management.

## Conclusions

We reported an incidental finding of asymptomatic right IJV agenesis and compensatory contralateral left IJV enlargement. Despite the risk of venous thrombosis and disruption of cerebral drainage, a decision was made to cannulate the left IJV given the nature of the surgical procedure and the necessity of fluid administration and hemodynamic monitoring. No complications were encountered intra- and postoperatively. This case highlights the critical role of routine precannulation ultrasound evaluation to identify rare but clinically significant vascular anomalies and to guide safe anesthetic management.
